# Supplementary figures and images for: Generation and characterization of a Meflin-CreERT2 transgenic line for lineage tracing in white adipose tissue
Source: PLoS One. 2021 Mar 24;16(3):e0248267. doi: 10.1371/journal.pone.0248267 (PMC7990287; doi:10.1371/journal.pone.0248267)

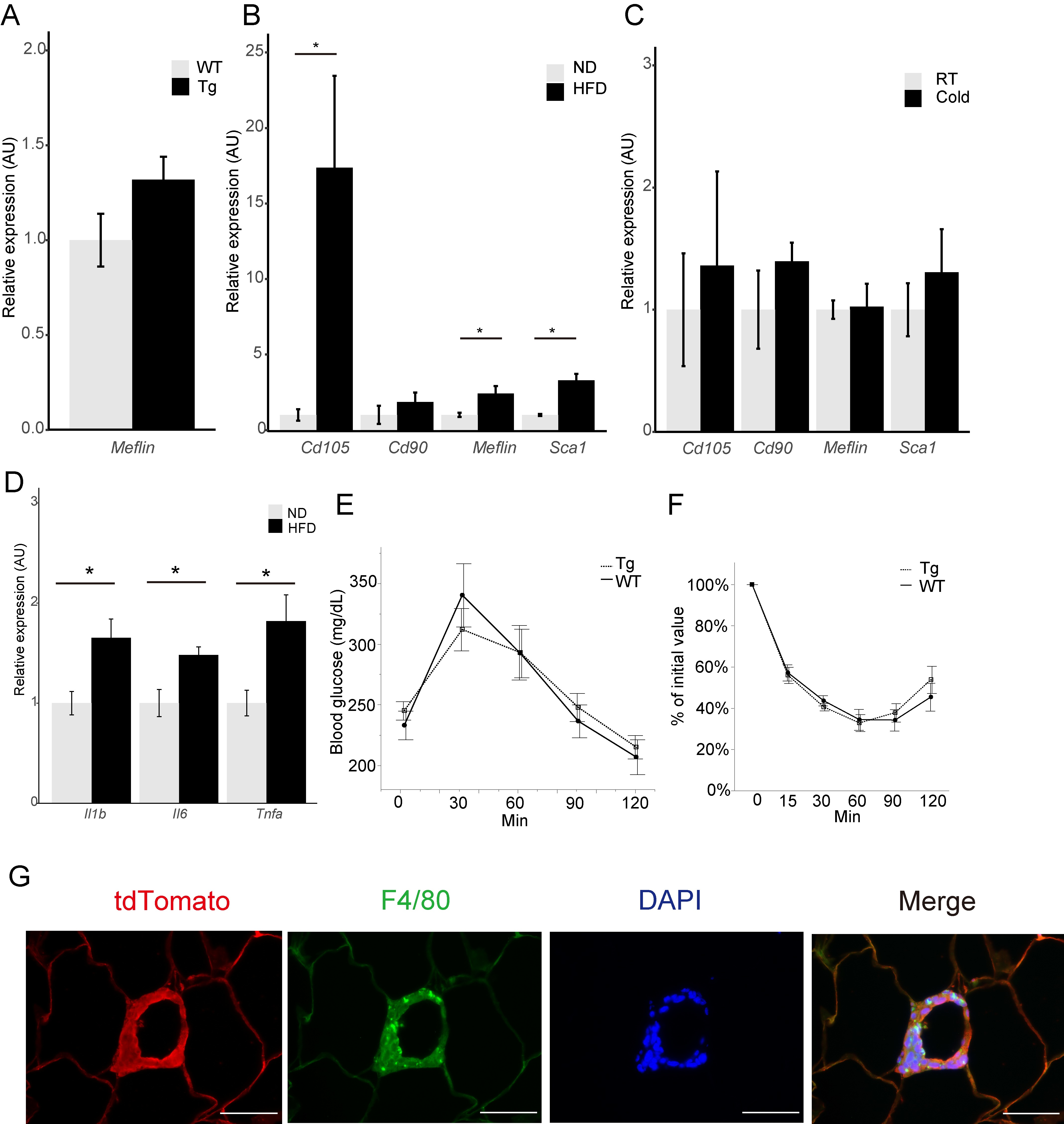

Supplement: S1 Fig — A. Meflin mRNA levels in the gonadal WAT (n = 4–5). B. QPCR of mesenchymal stem cell related genes in gonadal white adipose tissues of mice treated with normal diet (ND) or high-fat diet (HFD) for 8weeks (n = 4–5). *p < 0.05 by unpaired, 2-tailed t test. C. QPCR of mesenchymal stem cell related genes in inguinal white adipose tissues of mice treated with room temperature (RT) or cold stimulation (Cold) (n = 3–4). D. QPCR of inflammation related genes in gonadal white adipose tissues of mice treated with normal diet (ND) or high-fat diet (HFD) for 8weeks (n = 4–5). E. Oral glucose tolerance test (OGTT) of HFD-fed mice for 8 weeks with the transgenic allele (n = 6) or WT allele (n = 5). F. Insulin tolerance test (ITT) of HFD-fed mice for 8 weeks with the transgenic allele (n = 3) or WT allele (n = 4). G. The Crown like structure were consisted of the cells expressing tdTomato and F4/80. Meflin-CreERT2/Rosa26-tdTomato mice were fed a high-fat diet for 8 weeks and gonadal WAT (gWAT) samples were examined. Meflin lineage cells expressed tdTomato (red) and F4/80 (green). F4/80 is a mature macrophage marker. Nuclei were stained by DAPI (blue). Scale bars represent 50 μm. (TIF) [file pone.0248267.s001.tif]

Uncropped image used in Fig 1B

P1, P2, P8, P10, C1, C2, C3, C6

4.9 kb—  
2.9 kb—

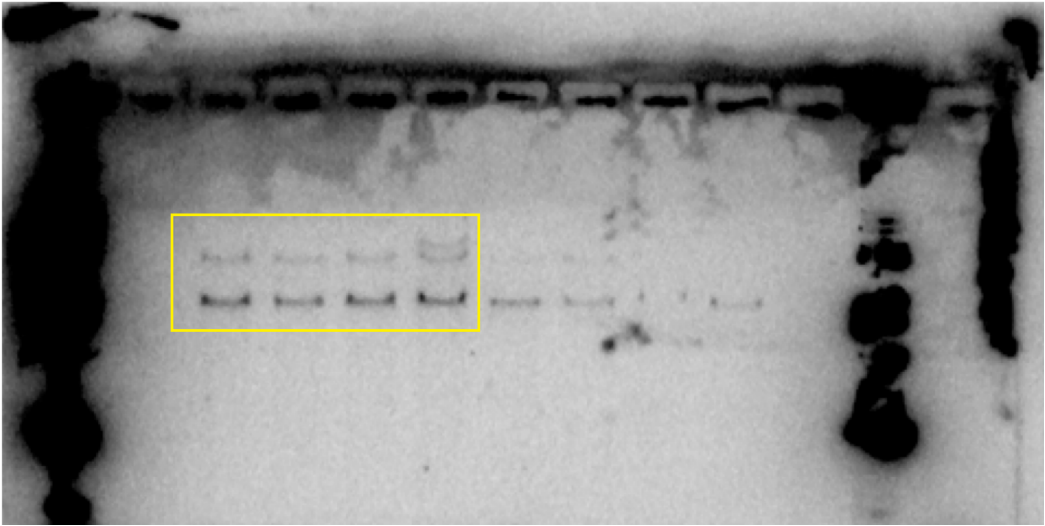

Supplement: S1 Raw images — (PDF) [file pone.0248267.s003.pdf]
