# Supplementary material for: Generation and characterization of a Meflin-CreERT2 transgenic line for lineage tracing in white adipose tissue
Source: PLoS One. 2021 Mar 24;16(3):e0248267. doi: 10.1371/journal.pone.0248267 (PMC7990287; doi:10.1371/journal.pone.0248267)
Supplement: S1 Table — (PDF) [file pone.0248267.s002.pdf]

Name	direction	sequence
Meflin	Forward	AGATCCGCTCGGTGGCTATT
Meflin	Reverse	AGGTCGCTCCAGGCAAACT
Cd105	Forward	CCCTCTGCCCATTACCCTG
Cd105	Reverse	GTAAACGTCACCTCACCCCTT
Cd90	Forward	TGCTCTCAGTCTTGCAGGTG
Cd90	Reverse	TGGATGGAGTTATCCTTGGTGTT
Sca1	Forward	TCTGAGGATGGACACTTCTC
Sca1	Reverse	CTCAGGCTGAACAGAAGCAC
Il1b	Forward	GCAACTGTTCCTGAACTCAACT
Il1b	Reverse	ATCTTTTGGGGTCCGTCAACT
Il6	Forward	TAGTCCTTCCTACCCCAATTTCC
Il6	Reverse	TTGGTCCTTAGCCACTCCTTC
Tnfa	Forward	ACGGCATGGATCTCAAAGAC
Tnfa	Reverse	AGATAGCAAATCGGCTGACG
Tbp	Forward	GAAGCTGCGGTACAATTCCAG
Tbp	Reverse	CCCCTTGTACCCTTCACCAAT
